# Gynecological cancer survivors with higher levels of physical and psychological symptoms consume less fruits and vegetables

**DOI:** 10.1093/abm/kaaf008

**Published:** 2026-03-13

**Authors:** Joann Kiebach, Sandra Beijer, Belle H de Rooij, Johanna M A Pijnenborg, M Caroline Vos, Dorry Boll, Roy F P M Kruitwagen, Nicole P M Ezendam

**Affiliations:** Department of Research and Development, Netherlands Comprehensive Cancer Organisation (IKNL), Boven Clarenburg 2, 3511CV, Utrecht, The Netherlands; Department of Health Evidence, Radboud University Medical Center, Houtlaan 4, 6525XZ, Nijmegen, The Netherlands; Department of Research and Development, Netherlands Comprehensive Cancer Organisation (IKNL), Boven Clarenburg 2, 3511CV, Utrecht, The Netherlands; Department of Research and Development, Netherlands Comprehensive Cancer Organisation (IKNL), Boven Clarenburg 2, 3511CV, Utrecht, The Netherlands; Department of Medical and Clinical Psychology, Tilburg University, Warandelaan 2, 5037AB, Tilburg, The Netherlands; Department of Obstetrics and Gynecology, Radboud University Medical Center, Houtlaan 4, 6525XZ, Nijmegen, The Netherlands; Department of Obstetrics and Gynecology, Elisabeth-TweeSteden Hospital, Dr. Deelenlaan 5, 5042AD, Tilburg, The Netherlands; Department of Gynecology, Catharina Hospital, Michelangelolaan 2, 5623EJ, Eindhoven, The Netherlands; Department of Obstetrics and Gynecology, School for Oncology and Reproduction, Maastricht University Medical Center, P. Debyelaan 25, 6229HX, Maastricht, The Netherlands; GROW-School for Oncology and Developmental Biology, Maastricht University, Minderbroedersberg 4-6, 6211LK, Maastricht, The Netherlands; Department of Research and Development, Netherlands Comprehensive Cancer Organisation (IKNL), Boven Clarenburg 2, 3511CV, Utrecht, The Netherlands; Department of Medical and Clinical Psychology, Tilburg University, Warandelaan 2, 5037AB, Tilburg, The Netherlands

**Keywords:** fruit, vegetables, symptoms, cancer survivors, theory of planned behavior

## Abstract

**Background:**

Low fruit and vegetable consumption among gynecological cancer survivors is associated with worse health outcomes, but this association could be bidirectional.

**Purpose:**

To investigate the association between physical and psychological symptoms and fruit and vegetable consumption, alongside a potential mediation by behavioral constructs of the theory of planned behavior (TPB).

**Methods:**

Self-reported data from a Dutch cohort of endometrial and ovarian cancer survivors (*n* = 227) was used. Physical and psychological symptoms were assessed at 12 months, TPB constructs, fruit and vegetable consumption at 18 months posttreatment. Associations of these symptoms with daily fruit (pieces) and vegetable (grams) consumption, and the role of intention as TPB construct were evaluated using mediation analyses. If mediation was present, successive regression analyses assessed possible involvements of distal TPB constructs (attitude, subjective norm, perceived behavioral control (PBC)).

**Results:**

About 53% and 32% of the survivors met the fruit or vegetable guidelines, respectively. Higher anxiety and depressive symptoms were directly associated with lower fruit consumption (*P* = .02). There were direct associations between higher fatigue or depressive symptoms and lower vegetable consumption (*P* < .05), partly mediated by intention (*P* = .01, *P* < .05, respectively). Of all distal TPB constructs, PBC was a possible mediator. More gastrointestinal symptoms showed a total association with lower vegetable consumption (*P* = .02).

**Conclusions:**

Gynecological cancer survivors with more physical and psychological symptoms consumed less fruits and/or vegetables. For vegetables, this was partly mediated by decreased intention and PBC. Dietary advice focusing on increasing intention and diminishing perceived barriers may empower survivors to integrate fruits and vegetables in their diet.

## Introduction

Endometrial and ovarian cancer affected approximately 417 000 and 314 000 women worldwide in 2020, respectively.^1^ The incidence of endometrial cancer has more than doubled over the past 3 decades, which is mainly attributed to advanced life expectancy in the aging population and increased rates of overweight.^2^ In contrast, ovarian cancer incidence has strongly decreased in the 20th century due to the use of oral contraception, but it has stabilized since.^3^ Unfortunately, the rather nonspecific symptoms of ovarian cancer often result in advanced stage diagnosis, leading to poor survival.^4^

A healthy diet, including sufficient fruit and vegetable consumption has been linked to lower mortality rates among ovarian cancer survivors.^5^ Furthermore, higher intakes of fruits and vegetables may reduce overweight and obesity.^6^ This is especially relevant for endometrial cancer patients, as around 40% of this population is obese,^7^ which is associated with comorbidities as well as reduced overall survival.^8^–11 The World Cancer Research Fund/American Institute for Cancer Research (WCRF/AICR) guidelines recommend cancer survivors to consume at least 5 portions or 400 g of fruits and vegetables per day.^12^ This underlines the importance of an adequate fruit and vegetable intake among survivors of endometrial and ovarian cancer.

Fruit and vegetable consumption has been associated with several symptoms of psychological, such as fatigue and depression, both in populations with and without cancer,^13^–16 and lifestyle interventions have already shown beneficial effects on the severity of these symptoms.^17^,18 However, the link between fruit and particularly vegetable consumption and these outcomes could likely also be bidirectional, since cancer survivors that are suffering from fatigue and depression might not have the energy to spend much time on preparing healthy meals. This assumption is supported by the cross-sectional character of many studies postulating this connection,^13^ since they do not allow conclusions on the direction of the association.

The theory of planned behavior (TPB) is a behavioral model used to understand motivation for performing behaviors, and has also been applied to health behaviors.^19^,20 For cancer survivors, the model is especially relevant, since the social cognitive factors it includes (affective, normative, and control) may all be influenced by side effects of cancer treatments.^21^ According to the TPB, the direct or “immediate” determinant of a behavior is intention, ie, the motivational factors, or how much effort people are willing to make to perform this behavior. Intention again is influenced by 3 main prior constructs: (1) the attitude towards the behavior, thus a positive or negative evaluation, (2) the subjective norm, or the perceived social pressure to perform the behavior, and (3) the perceived behavioral control (PBC), which refers to the control and confidence over performing the behavior.^22^ The constructs of TPB predicted fruit and vegetable consumption well among healthy adults of different age groups.^23^–27 Nevertheless, in cancer populations, evidence on the use of the TPB or other behavioral models to explain fruit and vegetable consumption is limited. One study by Andrykowsky and colleagues investigated the association between the TPB constructs and different health behaviors, including fruit and vegetable consumption, among cancer survivors of various tumor types.^28^ They found an association between attitude as only TPB construct and the intention of eating a healthy diet, while the actual intake was not assessed. However, up till now, no study has applied a combination of physical and psychological factors with social cognitive factors to gyneco-logical cancer survivors.

In a survey among breast cancer survivors, fatigue was reported as the strongest barrier to healthy eating, followed by depression.^29^ But also other symptoms, especially caused by cancer treatment, could influence dietary patterns. These include taste changes, loss of appetite,^30^ and gastrointestinal complaints like constipation, bloating, diarrhea, fecal incontinence, and nausea.^29^,31 Thus, among gynecological cancer survivors, a low consumption of fruits and vegetables may not only exacerbate disease-related factors, but also be caused by them. However, knowledge on this potential vicious circle, which might diminish overall health and quality of life, remains limited.

Therefore, this study aims to (1) assess the association between physical and psychological symptoms and fruit and vegetable consumption, (2) assess whether this association is mediated by intention as part of the TPB in a longitudinal sample, and (3) investigate which of the distal TPB variables (attitude, subjective norm, PBC) may play a role in this mediation. Since most of these variables naturally interfere with performing tasks of daily living, we hypothesized that they likely affect the control construct more than personal or normative factors. Understanding the underlying behavioral and cognitive factors, as assessed by the TPB, that keep gynecological cancer survivors from eating sufficient fruits and vegetables as part of a healthy diet is crucial to develop effective and long-lasting lifestyle interventions.

## Methods

### Design

 This study is a secondary, longitudinal analysis of data from the [Study name removed for masked review.], a pragmatic, cluster randomized controlled trial, conducted in 12 hospitals in the south of the Netherlands [Reference moved for masked review.]. The aim of the original trial was to study the effect of providing gynecological cancer patients with a Survivorship Care Plan to improve the provision of information and posttreatment care. The plan provided only little information on lifestyle, and there was no difference in fruit and vegetable consumption between trial arms [data not shown]. Therefore, data of both arms were analyzed together as a prospective cohort. In this study, data were used from baseline (after surgery; descriptive and clinical characteristics), at 12 months follow-up (physical and psychological symptoms) and 18 months follow-up (fruit/vegetable consumption; TPB determinants). The medical research ethics committees of the participating hospitals approved the study [Reference moved for masked review.]. The current study followed the Strengthening the Reporting of Observational Studies in Epidemiology (STROBE) Statement reporting of cross-sectional studies.^32^

### Participants and data collection

To be eligible for study participation, patients had to (1) be newly diagnosed with either primary endometrial or ovarian cancer between April 2011 and March 2014, (2) undergo curative treatment, (3) be aged ≥18 years, and (4) be able to complete a Dutch questionnaire.

### Measurements

#### Fruit and vegetable consumption and TPB determinants

Fruit and vegetable consumption was assessed using a food-frequency format. Participants reported on how many days per week, (0-7 days) and how many portions per day (1-5 or more) they usually consume fruit, warm vegetables (eg, cooked, baked), and salad (eg, lettuce, cucumber, tomato etc.) individually. For fruit consumption, 1 portion corresponds to 1 piece or 1 serving of fruit. For vegetable consumption, 1 portion corresponds to 1 serving spoon of 50 g. Average daily consumption was calculated as: (consumption days*portions per day)/7. Participants were considered to meet the Dutch guidelines of fruit or vegetable consumption if they consumed on average at least 2 pieces of fruit or 4 serving spoons of vegetables per day, respectively.^33^ At the time of the study the guideline for vegetable consumption was 4 serving spoons, while currently it is 5.^34^ Theory of planned behavior constructs were assessed for fruit and vegetable consumption separately in a similar manner. On 5-point scales, participants could indicate, if they find eating 2 pieces of fruit or 200 g of vegetables daily important (attitude; “very unimportant”-“very important”), if people around them find their consumption important (subjective norm; “yes, for sure”-“no”), if they find it difficult to eat this amount (PBC; “very difficult”-“very easy”), and if they plan to eat the abovementioned amount daily in the upcoming 6 months (intention; “yes, for sure”-“no”).

#### Fatigue

Fatigue was measured with the Fatigue Assessment Scale (FAS).^35^ The FAS has been validated in a variety of populations including cancer survivors,^36^ and includes a total of 10 items, with 5 items each representing physical and mental fatigue. Items are scored from 1 (never) to 5 (always). A total score was calculated as the sum of all 10 items, ranging from 10 to 50. To assess whether clinically relevant fatigue was present, the scale was dichotomized into “no fatigue” (scores 10-21) and “fatigue” (scores 22-50), based on previous studies.^37^,38

#### Depressive symptoms and anxiety

Depressive symptoms and anxiety were assessed using the Hospital Anxiety and Depression Scale,^39^ which is validated in cancer populations.^40^,41 It consists of 14 items, of which 7 relate to depressive symptoms and 7 to anxiety. Each item is scored from 0 to 3, where 3 indicates a state that corresponds to the worst depressive symptoms or anxiety. All 7 scores were summed up to the total depressive symptoms or anxiety subscale, respectively, ranging from 0 to 21. A cutoff score of 8 was used to indicate depressive symptoms or anxiety.^39^,^41^

#### Gastrointestinal symptoms

Gastrointestinal symptoms were assessed using the gastrointestinal symptom scores of the validated European Organization for Research and Treatment of Cancer Quality of Life Questionnaire (EORTC QLQ) modules for endometrial (QLQ-EN24)^42^,^43^ and ovarian (QLQ-OV28)^44^ cancer. Each score contained 6 abdominal and bowel-related items with answers given on a 4-point Likert scale (“not at all” to “very much”). According to the EORTC scoring manual, the raw scale scores calculated from these items as average was transformed linearly into the total score for gastrointestinal symptoms, with a scale ranging from 0 to 100 and higher scores indicating higher symptom severity.^45^

#### Clinical and sociodemographic characteristics

Clinical characteristics were obtained from the Netherlands Cancer Registry including cancer type (endometrial/ovarian), tumor stage (I-IV), and type of treatment (surgery, radio-therapy, chemotherapy). Self-administered questions included age (at time of questionnaire), educational level (low [no or primary school], intermediate [lower general secondary education/vocational training], high [high vocational training/ university]) and marital status (partner/no partner). Body Mass Index (BMI; weight in kg/height in m²) was also based on self-report.

### Statistical analyses

Statistical analyses were performed using R Studio (version 4.0.2). A *P*-value < .05 was considered statistically significant. Characteristics of the participants were described as numbers with percentages, or medians with interquartile ranges, since parameters were not normally distributed. The data were presented for the total population and stratified for meeting the guidelines for fruit or vegetable consumption. To assess differences in characteristics between the participants, Wilcoxon rank sum tests, Chi-squared texts (χ^2^) and Fisher exact test were used.

For the subsequent analyses, missing data was imputed using multiple imputation (“mice - multivariate imputation by chained equations” package in R) with predictive mean matching for continuous and count data, logistic regression for binary and a proportional odds model for categorical data. Average missingness per variable was 3%. In total, 5 datasets were created with 10 iterations performed for each variable. Convergence of the imputations was inspected using trace plots. Analyses were performed with all datasets and subsequently, results were pooled. For all analyses, the TPB variables were dichotomized by grouping the lower 3, as well as the upper 2 answers of the 5-point scale to be able to use logistic regression, since they were not normally distributed, and a transformation was not successful.

Mediation analyses were performed to assess whether the intention for consumption mediated a potential association between physical and psychological symptoms (fatigue, depressive symptoms, anxiety, gastrointestinal symptoms) and vegetable and fruit consumption, respectively. Since attitude, subjective norm and PBC are thought to be direct precursors and to converge into intention, intention was chosen as proxy of the TPB constructs in the analysis. The direct association describes the adjusted association between the physical or psychological symptom variable and consumption without including intention, while the indirect association refers to the part of the association that passes through intention. The sum of both associations is referred to as total association. Causal mediation analyses were performed with the *CMAverse* package in R studio using a regression-based approach,^46^,^47^ to obtain point estimates of causal effects. For vegetable consumption, the analysis was based on linear regression and estimates are expressed as betas indicating changes in grams of vegetable consumption. Since fruit consumption could not be considered as continuous but as count data, Poisson regression was used, and estimates were expressed as rate ratios indicating a % change in fruit consumption. Potential preidentified confounders were age (years; continuous), education (low/medium/high; categorical), partner status (partner/no partner; categorical) and BMI (kg/m², continuous), since these have been associated with vegetable and fruit consumption as well as depressive symptoms, anxiety, and fatigue.^48^–^52^ Potential confounders for the association between gastrointestinal symptoms and vegetable or fruit in-take were BMI and age.^53^ Bootstrap resamples (*n* = 999) were taken to obtain standard errors and confidence intervals of the causal effects. Preferably, these analyses would have been performed using 3 separate timepoints, but due to our study design we had to use data on TPB constructs and fruit and vegetable consumption assessed at the same timepoint.

For associations where mediation was present, successive regression analyses were performed to describe how the more distal TPB variables (attitude, subjective norm, PBC) were involved in the association. For this, 5 multiple regressions were conducted for each significant symptom variable separately. Firstly, fruit or vegetable consumption was regressed on the symptom variable as well as all TPB variables using Poisson or linear regression, respectively. Further, intention was regressed on the symptom as well as the more distal TPB variables, and lastly, attitude, subjective norm and PBC were each regressed on the symptom variable, using lo-gistic regression. All associations were tested including the abovementioned confounders, but only significant associ¬ations were included in the final diagrams. Variable inflation factors (VIFs) were tested for the models, but no indication of multicollinearity was found (all VIFs below 3). For the linear regression, *R*^2^ were computed, while for the logistic and Poisson regression, pseudo *R*^2^ were calculated using Aldrich-Nelson pseudo *R*^2^ with Veall-Zimmermann correction. This method was chosen because it has the closest correspondence with ordinary *R*^2^ measures,^54^ to facilitate the comparability of the results. Because the software did not allow for pooled calculation of the pseudo *R*^2^ with the imputed datasets, all *R*^2^ were reported based on the nonimputed data.

## Results

### Participant characteristics

**Figure 1.**
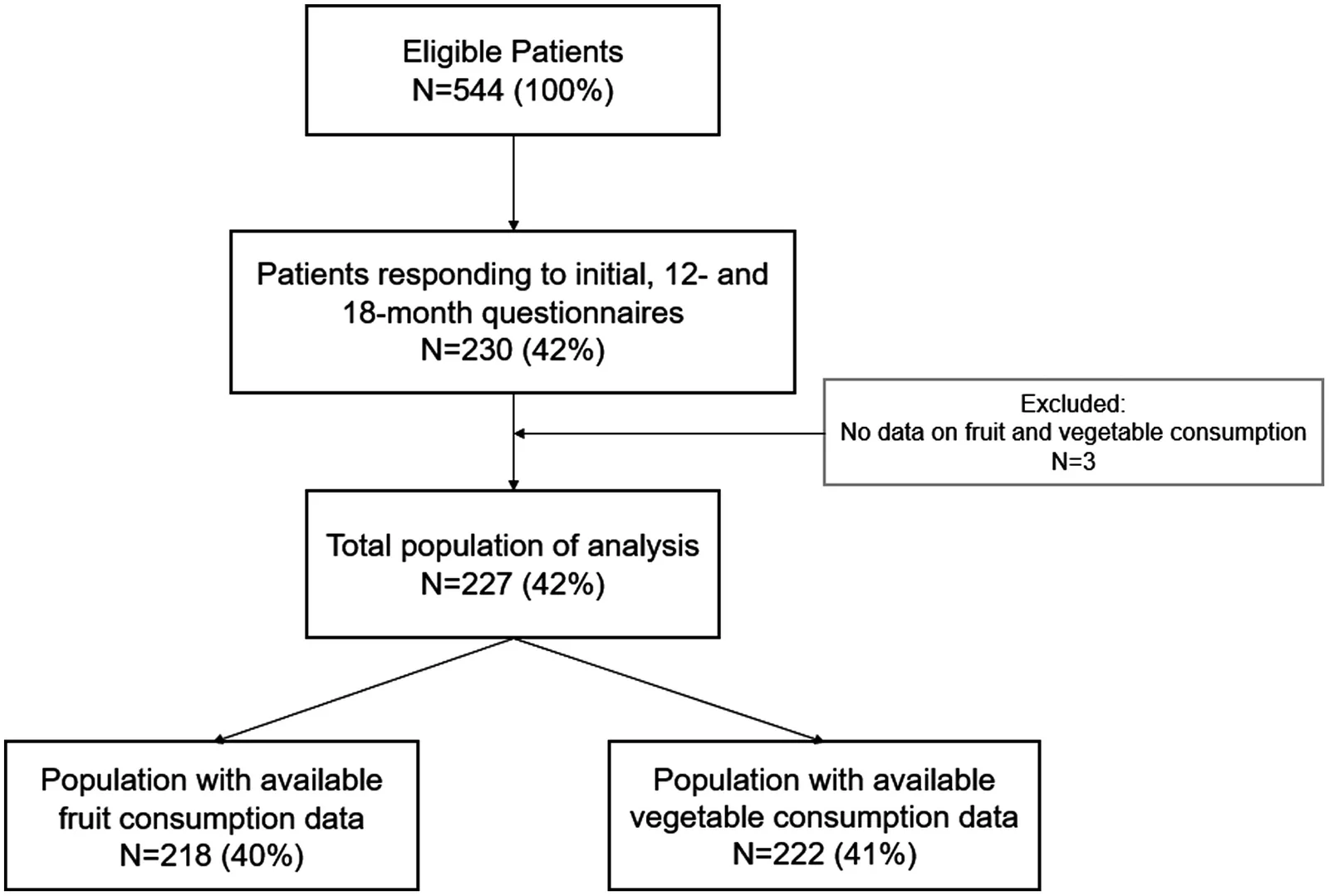
Flow diagram of gynecological cancer patients and participant selection through the study.

In total, 544 participants (n = 296 endometrial, *n* = 248 ovarian) were eligible, and 73% of the participants re¬sponded to the first questionnaire directly after initial sur¬gery (*n* = 395). Response rates at 12 and 18 months were 46% (*n* = 248) and 42% (*n* = 230), respectively. Of those re-sponding at 18 months, 3 participants were excluded from the analyses due to missing data for both fruit and vegetable consumption (Figure 1).

Participants had a median age of 67 years (IQR 60-72, range 25-80), and the majority (71%) had an intermediate educational level, used as proxy for socioeconomic status [not available] in this study. Endometrial cancer was diag¬nosed among 60% of participants, and 63% had 2 or more comorbidities (Table 1). Most participants had stage I disease (72%). Forty-four percent of participants reported fatigue at 12 months after diagnosis, while 27% and 14% reported anxiety or depressive symptoms, respectively.

**Table 1.** Characteristics of gynecological cancer survivors stratified by fruit and vegetable intake.

		Fruit consumption (*N* = 218)			Vegetable consumption (*N* = 222)		
	Total *N* = 227	Not meeting guidelines *N* = 102	Meeting guidelines^a^ *N* = 116	P-value^c^	Not meeting guidelines *N* = 151	Meeting guidelines^b^ *N* = 71	P-value^c^
Age	67 [60-72]	66 [58-72]	67 [61-72]	.24	67 [61-73]	64 [58-70]	.02*
Educational level^d^							
Low	29 (13)	12 (12)	17 (15)	.82	22 (15)	7 (10)	.40
Intermediate	159 (71)	72 (72)	79 (69)		105 (71)	49 (70)	
High	35 (16)	16 (16)	18 (16)		21 (14)	14 (20)	
Missing	4	2	2		3	1	
Partner status				.82			.78
Partner	168 (78)	79 (80)	84 (79)		114 (79)	50 (77)	
No partner	47 (22)	20 (20)	23 (21)		31 (21)	15 (23)	
Missing	12	3	9		6	6	
BMI (kg/m2)	27.4 [24.1-32.9]	28.1 [24.8-33.9]	26.1 [24.0-32.0]	.11	27.6 [24.3-32.8]	26.3 [24.0-33.6]	.65
Missing	16	3	13		7	9	
Cancer type							
Endometrial	137 (60)	62 (61)	70 (60)	.95	92 (61)	44 (62)	.88
Ovarian	90 (40)	40 (39)	46 (40)		59 (39)	27 (38)	
Stage							
I	152 (72)	73 (76)	74 (70)	.55	99 (70)	51 (78)	.22
II	9 (4.3)	2 (2.1)	6 (5.7)		5 (3.5)	4 (6.2)	
III	37 (18)	15 (16)	20 (19)		27 (19)	9 (14)	
IV	12 (5.7)	6 (6.2)	6 (5.7)		10 (7.1)	1 (1.5)	
Missing	17	6	10		10	6	
Treatment							
Surgery	221 (98)	100 (99)	112 (97)	.62	147 (98)	70 (100)	.55
Surgery + adjuvant therapy	112 (50)	41 (41)	64 (56)	.03*	81 (54)	27 (39)	.03*
Missing	2	1	1		1	1	
Comorbidities							
0	23 (11)	7 (7.2)	13 (12)	.47	14 (9.8)	9 (14)	.45
1	55 (26)	27 (28)	26 (24)		35 (24)	19 (29)	
2 or more	135 (63)	63 (65)	68 (64)		94 (66)	37 (57)	
Missing	14	5	9		8	6	
EORTC Gastrointestinal symptomse	11 [6-22]	11 [6-22]	11 [6-22]	.65	17 [6-22]	11 [0-17]	.02*
Missing	11	4	7		5	6	
FAS fatigue							
Score	20 [17-26]	21 [17-26]	20 [17-26]	.25	21 [17-27]	19 [16-23]	.03*
Fatigued (yes)	96 (44)	46 (46)	47 (43)	.59	71 (48)	22 (34)	.06
Missing	9	3	6		3	6	
HADS anxiety							
Score	5 [2-8]	6 [2-9]	4 [2-7]	.06	5 [2-8]	3 [1-7]	.11
Anxiety (yes)	59 (28)	35 (35)	24 (22)	.03*	43 (29)	14 (22)	.25
Missing	9	3	6		3	6	
HADS depressive symptoms							
Score	3 [1-6]	3 [1-6]	2 [1-6]	.22	3 [1-6]	2 [0-4]	.01*
Depressive symptoms	31 (14)	16 (16)	13 (12)	.38	23 (16)	4 (6.2)	.06
Missing	10	3	7		4	6	
Meeting guidelines (yes)							
Fruit	116 (53)	–	–	–	65 (45)	49 (71)	.001*
Missing	9				7	2	
Vegetables	71 (32)	20 (20)	49 (43)	.001*	–	–	–
Missing	5	3	2				

Data are presented as median [IQR, Q1-Q3] or number (percentage). Statistically significant P-values are indicated by asterisks (*). Missingness was indicated below each variable, unless there were no missing observations. ^a^Meeting Dutch dietary guidelines (eating on average at least 2 pieces of fruit daily). ^b^Meeting Dutch dietary guidelines (eating on average at least 200 g vegetables daily). ^c^P-values: comparison between meeting vs not meeting fruit or vegetable guidelines according to Wilcoxon rank sum test, Pearson’s chi-squared test, Fisher’s exact test. ^d^Educational levels are distinguished into low (no or primary school), intermediate (lower general secondary education/ vocational training) and high (high vocational training/university). ^e^According to the gastrointestinal symptom scale of the EORTC QLQ-EN24 or QLQ-OV28 for endometrial or ovarian cancer patients, respectively; higher score indicating higher symptom severity.

Out of the 218 participants with data on fruit consump¬tion, 53% (*N* = 116) reported to eat on average 2 or more pieces of fruit and thus, met the Dutch dietary guidelines. Those who met the guidelines on fruit consumption, received surgery with adjuvant therapy more often (56% vs 41%), and suffered less from anxiety (22% vs 35%).

Among the 222 participants reporting their vegetable con¬sumption, 71 (32%) met the dietary guidelines of at least 200 g of vegetables per day. Participants meeting the vege¬table consumption guidelines were younger (median 64 [IQR 58-70] vs 67 [IQR 61-63] years) and less often had adjuvant therapy (39% vs 54%) and had lower median gastrointestinal symptom scores (11 [IQR 0-17] vs 17 [IQR 6-22]), tended to be fatigued less often (34% vs 48%), and tended to report de¬pressive symptoms less frequently (6.2% vs 16%).

Lastly, participants meeting the fruit consumption guide¬lines more often also met the vegetable consumption guide¬lines (43% vs 20%) and vice versa, participants meeting the vegetable guidelines also met the fruit guidelines more fre¬quently (71% vs 45%).

Both fruit and vegetable consumption correlated with all of their corresponding TPB variables, with strongest cor¬relations for intention (*r* = 0.73 and 0.63, respectively) and weakest correlations for subjective norm (*r* = 0.32 and 0.19, respectively) (Supplementary Table 1).

### Mediation analyses for intention in the association between physical and psychological symptoms and fruit/vegetable consumption

There was a significant total association between fatigue and vegetable consumption (B -2.14 [CI, -3.53, -0.49]), indicating that a 1-point higher fatigue score was associated with a vegetable consumption of around 2 g less per day (Table 2). A direct association of B -1.68 [CI, -3.16, -.06] and an indirect association of B -0.46 [CI, -0.59, -0.20] were observed. Overall, 22% of the association between fatigue and vegetable consumption were mediated by intention. For depressive symptoms and vegetable intake, there was a significant total association (B −4.46 [CI, −7.73, −1,11]). The direct association of B −3.19 [CI, −6.19, −0.06] and the indirect association of B −1.28 [CI, −2.46, −0.08] were both significant, and 29% of the association between depressive symptom scores and vegetable consumption were mediated by intention. With respect to gastrointestinal symptoms, there was a significant total association (B −0.83 [CI, −1.61, −0.13]), while neither the direct nor the indirect association itself were significant. There was no significant association between anxiety and vegetable consumption.

**Table 2.** 

		Fruit consumption		Vegetable consumption	
Variable	*N* = 227*	Rate ratio	P-value	B	P-value
Fatigue^a,b,c,d^	Total association	0.99 [0.98, 1.01]	.30	−2.14 [−3.53, −0.49]	.01*
	Direct association	0.99 [0.98, 1.01]	.26	−1.68 [−3.16, −0.06]	.04*
	Indirect association	1.00 [1.00, 1.01]	.67	−0.46 [−0.59, −0.20]	.01*
	Proportion mediated	0.18 [−3.11, 4.40]	.55	0.22 [0.07, 0.02]	.02*
Anxiety^a,b,c,d^	Total association	0.98 [0.96, 1.01]	.13	−1.63 [−5.20, 0.65]	.15
	Direct association	0.98 [0.96, 0.998]	.02*	−1.09 [−4.21, 1.02]	.25
	Indirect association	1.00 [0.99, 1.02]	.68	−0.54 [−1.65, 0.75]	.35
	Proportion mediated	−0.15 [−3.86, 4.41]	.84	0.35 [−2.48, 2.19]	.37
Depressive symptoms^a,b,c,d^	Total association	0.98 [0.96, 1.01]	.12	−4.46 [−7.73, −1,11]	.01*
	Direct association	0.98 [0.96, 0.997]	.02*	−3.19 [−6.19, −0.06]	.046*
	Indirect association	1.00 [0.99, 1.02]	.94	−1.28 [−2.46, −0.08]	.044*
	Proportion mediated	0.98 [−3.69, 4.01]	.93	0.29 [−0.002, 0.94]	.052
Gastrointestinal symptomsa,c	Total association	1.00 [0.99, 1.00]	.42	−0.83 [−1.61, −0.13]	.02*
	Direct association	1.00 [0.99, 1.00]	.10	−0.57 [−1.35, 0.06]	.08
	Indirect association	1.00 [1.00, 1.01]	.64	−0.26 [−0.54, 0.11]	.17
	Proportion mediated	−0.37 [−7.60, 7.74]	.94	0.31 [−0.31, 1.08]	.21

Use of Poisson regression to determine associations with fruit consumption and linear regression to determine associations with vegetable consumption. Rate ratio indicates % change in fruit consumption. Mediation analyses were performed using *n* = 999 bootstrap resamples. Statistically significant P-values are indicated by asterisks (*).

*Data was imputed using multiple imputation with 10 iterations per imputation. Analyses were performed on 5 imputed datasets. Confounders:

^a^Age,

^b^Education,

^c^BMI,

^d^Partner status.

For fruit consumption, a significant direct association between anxiety (RR 0.98 [CI, 0.96, 0.998]), as well as depressive symptoms (RR 0.98 [CI, 0.96, 0.997]) and fruit consumption was observed (Table 2). This indicates that a 1-point increase in anxiety or depressive symptom scales, reduced the fruit consumption by 2%. However, there was no significant indirect association and thus, intention did not mediate these associations. Neither fatigue nor gastrointestinal symptoms were significantly associated with fruit consumption.

### Successive regression analyses for fatigue or depressive symptoms and vegetable consumption including distal TPB variables

**Figure 2.**
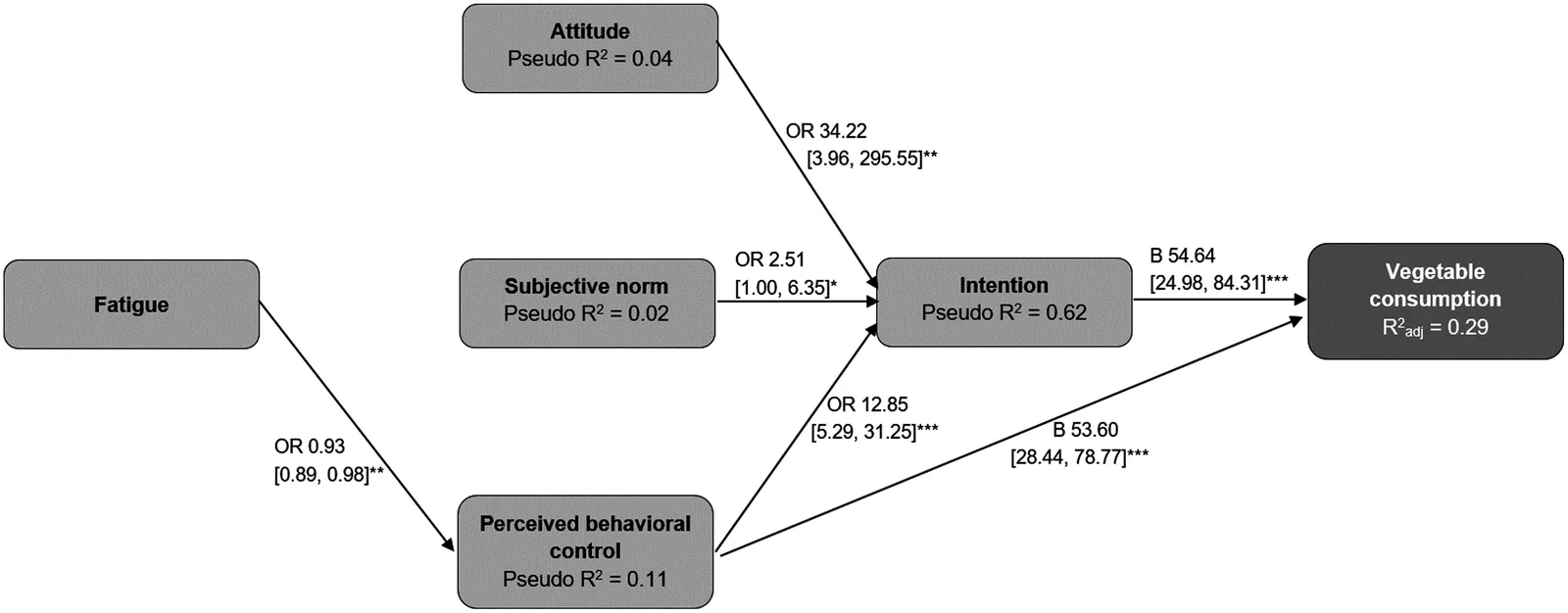
Association between fatigue scores, TPB constructs and vegetable consumption among gynecological cancer survivors (*n* = 227). Associations were assessed using linear regression for vegetable consumption (in g) or logistic regression for TPB variables and expressed as beta (B) or odds ratio (OR) with 95% confidence interval. Models were adjusted for age, education level, partner status and BMI. *, **, and *** represent a significance of *P* < .05, .01, and .001, respectively. For logistic regression models, Pseudo *R*^2^ were calculated using Aldrich-Nelson pseudo *R*^2^ with Veall-Zimmermann correction.

To better understand the indirect paths of fatigue and depressive symptoms with vegetable consumption, we conducted a sequence of regression analyses including all TPB constructs. After including all TPB items, fatigue was not directly associated with vegetable consumption (Figure 2). The *R*^2^ indicates that 29% of the variance in vegetable consumption were explained by the model. Intention and PBC had a significant direct association with vegetable consumption. Thus, a difference between low to high intention or PBC was associated with a mean difference in vegetable consumption of 55 g [CI, 25.0, 81.3] and 54 g [CI, 28.4, 78.8], respectively. There was a significant association between fatigue scores and PBC (OR 0.93 [CI, 0.89, 0.98]), showing that 1-point higher fatigue scores were related to 7% lower odds of having a high PBC.

**Figure 3.**
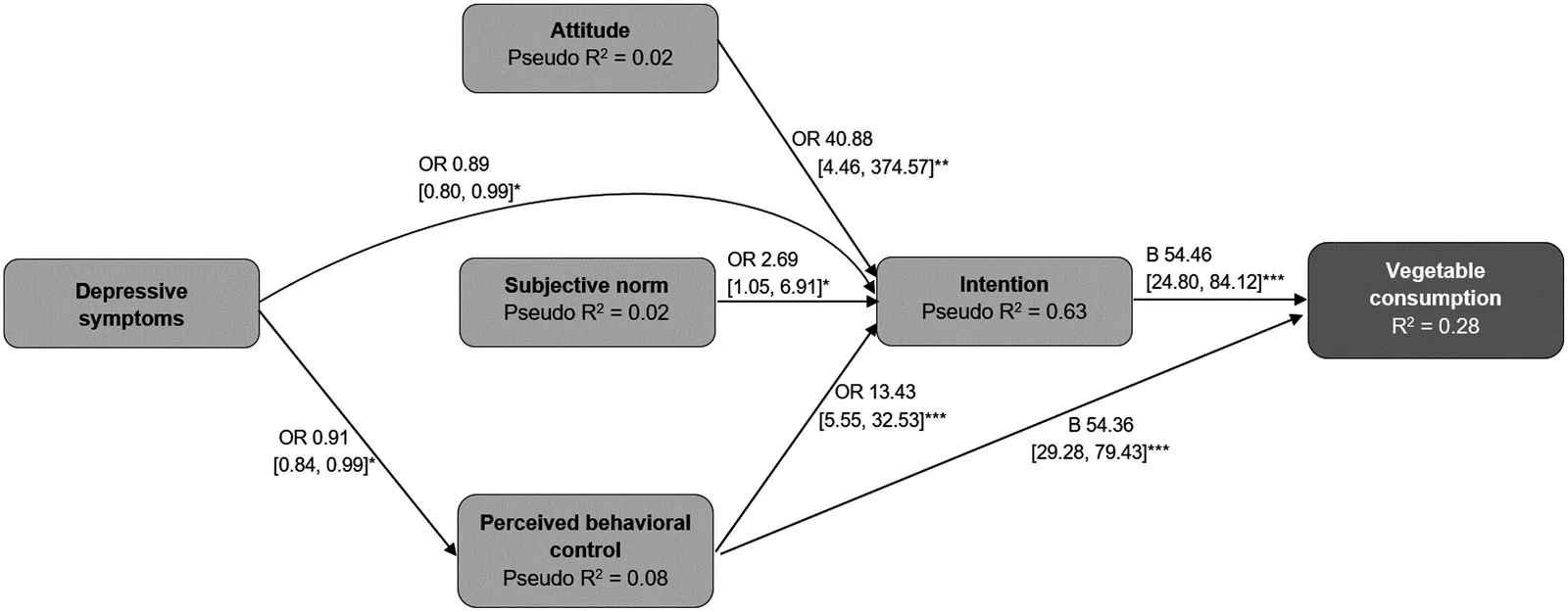
Association between depressive symptom scores, TPB constructs and vegetable consumption among gynecological cancer survivors (*n* = 227). Associations were assessed using linear regression for vegetable consumption (in g) or logistic regression for TPB variables and expressed as beta (B) or odds ratio (OR) with 95% confidence interval. Models were adjusted for age, education level, partner status and BMI. *, **, and *** represent a significance of *P* < .05, .01, and .001, respectively. For logistic regression models, Pseudo *R*^2^ were calculated using Aldrich-Nelson pseudo *R*^2^ with Veall-Zimmermann correction.

After including all TPB variables, depressive symptoms were not directly associated with vegetable consumption. Again, intention (B 54.5 [24.8, 84.1]) and PBC (B 54.4 [CI, 29.3, 79.4]) were significantly associated with vegetable consumption (*R*^2^ = 0.28; Figure 3). Depressive symptoms were significantly associated with PBC (OR 0.91 [CI, 0.84, 0.99]) and intention (OR 0.89 [CI, 0.80, 0.99]). Thus, a 1-point higher depressive symptoms score was associated with 9 and 11 times higher odds of having a high PBC and high intention, respectively.

## Discussion

This study showed that more than half of gynecological cancer survivors adhered to the fruit guidelines of 2 pieces per day, while less than a third met the dietary guidelines of eating at least 200 g of vegetables daily. Survivors with higher levels of anxiety and depressive symptoms were more likely to report lower fruit intake, which was not mediated by intentions. Regarding the consumption of vegetables, survivors who were more fatigued, had a higher level of depressive or gastrointestinal symptoms, consumed less vegetables compared to those with a lower symptom severity, which was partly mediated by decreased intentions and possibly also related to PBC for fatigue and depressive symptoms.

In previous studies among cancer survivors, adherence to fruit and vegetable guidelines varied. For fruits, we observed that 53% of the survivors met the guidelines, which is similar to 2 studies reporting adherence by 41% and 59% of ovarian and gynecological cancer populations, respectively.^^8^,^55^^ Regarding the vegetable guidelines, 32% of our population met these guidelines, while previous results vary between 29%^55^ and only 14%^8^ adherence. A Dutch study in colorectal cancer survivors, reported that fruit and vegetable guidelines were met by only 19% and 14% of the population, respectively.^14^ Compared to these previous studies, our population tended to have a similar adherence to the guidelines for fruit intake and slightly higher adherence for vegetable intake. These differences could be explained by the use of different guidelines in the studies, such as the WCRF/AICR recommendations (400 g fruits/vegetables per day),^^14^,^56^^ or the recommendation of eating 2 portions of fruits or 5 portions of vegetables daily,^8^ and consequently, different cutoffs were set for meeting the guidelines. Likely, the differences in adherence might also be due to different eating habits among survivor groups in different countries, as well as sex differences,^^14^,^57^^ and potentially higher gastrointestinal complaints in the case of colorectal cancer. Notably however, also in our study we observed that survivors with higher gastrointestinal symptom levels were less likely to meet the vegetable guidelines. Lastly, also the assessment methods differed between studies, and included single items from a national survey,^8^ a validated FFQ,^55^ and a 7-day dietary record.^14^ Especially the latter might explain the noticeably lower adherence in that study, since FFQs, as used in the current study, are more prone to overestimate intake than dietary records.^^58^,^59^^ Remarkably, in our study, as well as in previous research in cancer survivors, more people seemed to adhere to the fruit guidelines than to the vegetable guidelines. This suggests that it might be easier or more doable for survivors, but likely also noncancer populations,^60^ to eat a sufficient amount of fruits compared to vegetables, possibly because of the different ways of serving them. While fruits are frequently consumed as snacks, vegetables are mostly eaten as a warm dinner in the Dutch population, which requires more preparation time and effort.

Our study showed that higher levels of fatigue and depressive symptoms among gynecological cancer survivors were associated with lower fruit and vegetable consumption. To our knowledge, no previous study investigated the association between physical and psychological symptoms and fruit and vegetable consumption among gynecological cancer survivors in this direction. However, longitudinal studies among breast or colorectal cancer patients and a meta-analysis in the general population showed associations between healthy diet or fruit and vegetable consumption and a decrease in fatigue severity^^14^,^15^^ or depression risk.^16^ Similar to our findings, Kenkhuis et al. only found a relation between vegetable consumption and fatigue, but not for fruit consumption.^14^ In intervention studies, diets rich in fruits and vegetables reduced fatigue^18^ and depression^17^ significantly, compared to the control diets containing less fruits and vegetables. A survey looking at the same direction as our study (association between physical and psychological symptoms and health behaviors) identified that especially fatigue and depression are perceived as barriers to healthy nutrition among breast cancer survivors,^29^ which supported our results. Taken together, our findings and previous studies, which also include cross-sectional associations, suggest that the link between physical and psychological symptoms and the consumption of fruits and vegetables may be bidirectional and they possibly exacerbate each other.

In our study, anxiety and depressive symptoms were directly associated with fruit consumption, although the total effect, thus without consideration of the intention, was not significant. According to the classic mediation approach by Baron and Kenny,^61^ it would not be legitimate to conclude on a significant direct effect in this case. More recently however, consensus has grown among statisticians that direct or indirect effects in mediation analysis may still be valid despite a nonsignificant total effect.^62^ Therefore, higher anxiety and depressive symptoms might have a relation with a lower fruit consumption in our population, without being mediated my someone’s intention. Nevertheless, the intention to eat sufficient fruits was strongly positively related to the actual consumption. This suggests that even if survivors feel more anxious or have more depressive symptoms, this does not necessarily influence their intention to eat fruits. Possibly, other intrapersonal aspects play a role, such as capabilities and opportunities as suggested in other behavioral models.^63^ In this context, survivors with higher levels of anxiety or depressive symptoms might rather feel incapable of eating fruits or do not see opportunities to integrate this in their daily life, while they principally are still motivated, or have the intention to do so. Thus, it would be beneficial to also find underlying behavioral determinants and reasons for a lower fruit consumption among gynecological cancer survivors with higher levels of anxiety and depressive symptoms.

We observed a significant association between higher gastrointestinal symptoms and a lower vegetable consumption, which was not mediated by intention. Likely, survivors with these symptoms avoid certain vegetables that may further exacerbate complaints such as bloating,29 and therefore, their total intake decreases while their intentions to eat vegetables are rather unaffected. Adjuvant therapy is often assumed to cause bowel disturbances among gynecological cancer survivors, and especially radiation therapy may lead to long term gastrointestinal toxicities.^31^ Thus, adjuvant therapy may indirectly be related to a lower vegetable consumption among gynecological cancer survivors, and consequently, survivors should receive specific advice on how to maintain a good intake without increasing gastrointestinal problems, or possibly even decrease their complaints.

Vegetable consumption was lower among those who were more fatigued or had more depressive symptoms, which was partly mediated by their decreased intentions. In the TPB, intention is strongly related to motivation. Thus, higher fatigue and depressive symptom levels might affect the motivation of survivors to eat vegetables, partly because they seem to perceive less control over the consumption. Although a more favorable attitude was strongly related to intention, being more fatigued or having more depressive symptoms was not associated with attitude. This suggests that people seem indeed aware of the relevance of a sufficient vegetable consumption, but they might feel less in control and find it more difficult to prepare and eat vegetables. This is in line with the perception of fatigue and depressive symptoms as barriers to a healthy diet among cancer survivors.^29^ Taken together, the influence (all *P* < .001) of PBC and intention in the association between fatigue and depressive symptom levels and vegetable consumption could reflect that eating vegetables is perceived as burdensome, thus patients may feel too fatigued to spend time on cooking and, therefore, might lack the (perceived) capabilities and self-efficacy to integrate the preparation of dishes rich in vegetables in their daily routines.

To the best of our knowledge, this study showed for the first time that worse physical and psychological symptoms are associated with a lower fruit and vegetable consumption among gynecological cancer patients, and revealed that for vegetables, this association was partly mediated by decreased intentions. A study strength is that we assessed symptoms (at 12 months) before the assessment of the consumption as well as TPB constructs (at 18 months), which allows better conclusions on the direction of the associations and avoids the risk of reverse causation. Further, participants were recruited by population-based sampling, and we used validated assessments for fatigue, anxiety and depressive and gastrointestinal symptoms, which increases the validity of our results. Lastly, we used multiple data imputation to increase the statistical power of our analyses. Nevertheless, a limitation of our study is the rather small sample size, which did not allow us to stratify analyses by tumor type. However, tests for an interactive effect of tumor type in the association between physical and psychological symptoms and fruit or vegetable intake were nonsignificant. Furthermore, baseline characteristics regarding consumption and symptoms, except for gastrointestinal symptoms, were similar between endometrial and ovarian cancer survivors (Supplementary Table 2). Data on TPB determinants have been assessed simultaneously with the consumption of fruit and vegetables at 18 months. Although the questions regarding TPB constructs, apart from intention, refer to the present, we cannot exclude that this may have influenced our results, since the determinants should ideally precede the health behaviors. Unfortunately, this assessment schedule was inevitable due to the study design. In addition, the data of the TPB variables was not normally distributed, which limits the power of the analyses and made it impossible to do formal path analyses. Therefore, this data had to be dichotomized to nevertheless ensure appropriateness of analysis types. Another limitation is the use of self-reported food consumption data from an FFQ. This method is likely to show recall bias and/or socially desirable answers, and FFQs tend to overestimate consumption data compared to the actual intake.^58^ On the other hand, a more extensive self-reported method such as dietary records would have increased participant burden,^59^ and FFQs have shown to be at least sufficiently valid in ranking participants by intake.^58^ However, the definition of vegetables as either “warm,” or “salad” could possibly have led to a certain degree of underreporting in case patients consumed raw vegetables they did not consider as salad. Lastly, we used education as proxy for socioeconomic status, but not other measures such as income or occupation. Education is frequently used to measure socioeconomic status or inequalities in health research, but the use of additional variables may have given a more complete picture of the possible influence of socioeconomic position on outcomes.

Our study reveals PBC as a potential key starting point for increasing vegetable consumption in clinical practice. Given that higher fatigue and depressive symptom levels were directly associated with a lower PBC, it seems important to increase the sense of control of gynecological cancer survivors, thus, to increase the perceived access and easiness to preparing and eating healthy meals including sufficient vegetables. Thereby, eg, by giving options to the survivor for meals that are easy and fast to prepare, but still healthy and tasteful, counseling and follow-up care could improve vegetable consumption among cancer survivors. This could be especially important since intervention studies that successfully increased fruit and vegetable intake, showed to reduce fatigue and depressive symptoms, suggesting a bidirectional connection, so it might in turn even improve these symptoms in this population. This highlights the importance of dietary advice and support for facilitating fruit and especially vegetable consumption as part of the follow-up care of cancer survivors. In a Dutch cohort of colorectal cancer survivors, 62% reported they had been provided with nutritional information by healthcare professionals, which, next to dieticians also included nurses and doctors.^64^ By contrast, only 8% of gynecological cancer survivors within the [Study name removed for masked review.] reported to have received nutritional advice [Reference moved for masked review.]. Further, little is known about whether this nutritional information also includes behavioral aspects, such as support and empowerment for implementing a healthier diet. In light of our findings, this could be highly relevant, especially in specific populations like those with more symptoms of fatigue and depressive symptoms. These groups may already consider fruits and vegetable consumption important (since attitude was unrelated to symptoms) but may not feel “in charge” of consuming them sufficiently. Consequently, nutritional advice within follow-up care could, in addition to conveying knowledge, also help survivors to increase possibilities to easily integrate fruits and vegetables in their daily routine. Therefore, future intervention studies should investigate potential effects of dietary advice aiming at these behavioral aspects for fruit and vegetable consumption among different cancer populations. Since a healthy diet potentially decreases symptoms such as depression and fatigue,^^17^,^18^^ the aforementioned studies should also consider assessing the secondary effects of such an intervention on physical and psychological symptoms. Vice versa, due to the potentially bidirectional link, also studies on relieving these symptoms might consider changes in diet as an outcome. To develop such targeted dietary intervention studies, a review of existing interventions in cancer populations under consideration of behavioral theories may be necessary to evaluate the most effective strategies.

In conclusion, gynecological cancer survivors with stronger symptoms of fatigue, anxiety and depression, and gastrointestinal symptoms consumed less fruits and/or vegetables than those with less symptoms. For vegetable consumption, these associations were partly due to decreased intention and PBC. Our findings underline the need to identify survivors with these symptoms as risk groups for low fruit and vegetable consumption. Dietary advice within follow-up care should aim to reduce patients’ perceived barriers and empower them to integrate fruits and especially vegetables in their daily routine. Interventional studies in gynecologic and nongynecologic cancer populations, should assess effects of strategies to improve physical and psychological symptoms and to increase the PBC on fruit and vegetable consumption.

## Supplementary Material

kaaf008_Supplementary_Data

## Data Availability

De-identified data from this study are available in a protected archive: www.profilesregistry.nl. Data can be obtained by mailing the contact person using the contact information on the website. *Analytic code availability*: Analytic code used to conduct the analyses presented in this study are not available in a public archive. They may be available by emailing the corresponding author. *Materials availability*: Materials used to conduct the study are not publicly available.
