# Supplementary material for: Gynecological cancer survivors with higher levels of physical and psychological symptoms consume less fruits and vegetables
Source: Ann Behav Med. 2026 Mar 13;60(1):kaaf008. doi: 10.1093/abm/kaaf008 (PMC13374855; doi:10.1093/abm/kaaf008)
Supplement: kaaf008_Supplementary_Data [file kaaf008_supplementary_data.docx]

**Supplementary Table 1: correlations between fruit and vegetable consumption and their corresponding TPB variables.**

|  | Fruit consumption | Intention | Attitude | PBC | Norm |
| --- | --- | --- | --- | --- | --- |
| Fruit consumption |  |  |  |  |  |
| Intention | 0.73** |  |  |  |  |
| Attitude | 0.65** | 0.72** |  |  |  |
| PBC | 0.68** | 0.72** | 0.63** |  |  |
| Norm | 0.32** | 0.48** | 0.55** | 0.35** |  |
|  | **Vegetable consumption** | **Intention** | **Attitude** | **PBC** | **Norm** |
| Vegetable consumption |  |  |  |  |  |
| Intention | 0.63** |  |  |  |  |
| Attitude | 0.45** | 0.63** |  |  |  |
| PBC | 0.58** | 0.71** | 0.54** |  |  |
| Norm | 0.19* | 0.37** | 0.42** | 0.13* |  |

Correlations were calculated using Spearman’s rank correlation coefficient. * indicates a p-value <0.05 and ** indicates a p-value <0.001.

**Supplementary Table 2: Characteristics of gynecological cancer survivors stratified by tumor type**

|  |  | Cancer type | | |
| --- | --- | --- | --- | --- |
|  | Total  N = 227 | Endometrial cancer N = 137 | Ovarian cancer N = 90 | p-value^a^ |
| **Age** | 67 (60, 72) | 67 (63, 73) | 66 (57, 69) | 0.01* |
| **Educational level** |  |  |  |  |
| Low | 29 (13%) | 19 (14%) | 10 (11%) | 0.07 |
| Intermediate | 159 (71%) | 100 (75%) | 59 (66%) |  |
| High | 35 (16%) | 15 (11%) | 20 (22%) |  |
| Missing | 4 | 3 | 1 |  |
| **Partner status** |  |  |  |  |
| Partner | 168 (78%) | 104 (80%) | 64 (75%) | 0.41 |
| No partner | 47 (22%) | 26 (20%) | 21 (25%) |  |
| Missing | 12 | 7 | 5 |  |
| **BMI (kg/m²)** | 27.4 [24.1, 32.9] | 29.4 [25.6, 34.8] | 25.2 [23.3, 28.8] | 0.001* |
| Missing | 16 | 10 | 6 |  |
| **Stage** |  |  |  |  |
| I | 152 (72%) | 125 (94%) | 27 (35%) | 0.001* |
| II | 9 (4.3%) | 2 (1.5%) | 7 (9.1%) |  |
| III | 37 (18%) | 4 (3.0%) | 33 (43%) |  |
| IV | 12 (5.7%) | 2 (1.5%) | 10 (13%) |  |
| Missing | 17 | 4 | 13 |  |
| **Treatment** |  |  |  |  |
| Surgery | 221 (98%) | 137 (100%) | 84 (95%) | 0.02* |
| Surgery + adjuvant therapy | 112 (50%) | 45 (33%) | 67 (76%) | 0.001* |
| Missing | 2 | 0 | 2 |  |
| **Comorbidities** |  |  |  |  |
| 0 | 23 (11%) | 23 (18%) | 0 (0%) | 0.001* |
| 1 | 55 (26%) | 28 (22%) | 27 (31%) |  |
| 2 or more | 135 (63%) | 76 (60%) | 59 (69%) |  |
| Missing | 14 | 10 | 4 |  |
| **EORTC Gastrointestinal symptoms^b^** | 11 [6, 22] | 11 [6, 22] | 17 [6, 33] | 0.001* |
| Missing | 11 | 8 | 3 |  |
| **FAS fatigue** |  |  |  |  |
| Score | 20 [17, 26] | 20 [17, 24] | 21 [17, 29] | 0.11 |
| Fatigued (yes) | 96 (44%) | 53 (40%) | 43 (49%) | 0.19 |
| Missing | 9 | 6 | 3 |  |
| **HADS anxiety** |  |  |  |  |
| Score | 5.0 [2.0, 8.0] | 4.0 [1.0, 7.0] | 6.0 [3.0, 8.0] | 0.01* |
| Anxiety (yes) | 59 (27%) | 30 (23%) | 29 (33%) | 0.09 |
| Missing | 9 | 6 | 3 |  |
| **HADS depressive symptoms** |  |  |  |  |
| Score | 3.0 [1.0, 6.0] | 3.0 [1.0, 6.0] | 2.5 [1.0, 6.8] | 0.65 |
| Depressive symptoms (yes) | 31 (14%) | 17 (13%) | 14 (16%) | 0.50 |
| Missing | 10 | 6 | 4 |  |
| **Meeting guidelines (yes)** |  |  |  |  |
| Fruit^c^ | 116 (53%) | 70 (53%) | 46 (53%) | 0.95 |
| Missing | 9 | 5 | 4 |  |
| Vegetables^d^ | 71 (32%) | 44 (32%) | 27 (31%) | 0.88 |
| Missing | 5 | 1 | 2 |  |

*Data are presented as median [IQR, Q1-Q3] or number (percentage). Statistically significant p-values are indicated by asterisks (*).*

*^a^ p-values: comparison between meeting vs. not meeting fruit or vegetable guidelines according to Wilcoxon rank sum test, Pearson’s Chi-squared test, Fisher’s exact test.*

*^b^ according to the gastrointestinal symptom scale of the EORTC QLQ-EN24 or QLQ-OV28 for endometrial or ovarian cancer patients, respectively; higher score indicating higher symptom severity.*

*^c^ Meeting Dutch dietary guidelines (eating on average at least 2 pieces of fruit daily).*

*^d^ Meeting Dutch dietary guidelines (eating on average at least 200 g vegetables daily).*
